# Interplay between 3′-UTR polymorphisms in the methylenetetrahydrofolate reductase (*MTHFR*) gene and the risk of ischemic stroke

**DOI:** 10.1038/s41598-017-12668-x

**Published:** 2017-09-29

**Authors:** Jung Oh Kim, Han Sung Park, Chang Soo Ryu, Jung-Won Shin, Jinkwon Kim, Seung Hun Oh, Ok Joon Kim, Nam Keun Kim

**Affiliations:** 1 Institute for Clinical Research, CHA Bundang Medical Center, School of Medicine, CHA University, Seongnam, 463-712 South Korea; 2 Department of Biomedical Science, College of Life Science, CHA University, Seongnam, South Korea; 3 Department of Neurology, CHA Bundang Medical Center, School of Medicine, CHA University, Seongnam, 463-712 South Korea

## Abstract

Stroke incidence is a multifactorial disease and especially hyperhomocysteinemia is associated with a higher risk of stroke. Previous studies have reported a folate metabolism disorder associated with the *MTHFR* gene. We investigated four single nucleotide polymorphisms in the *MTHFR* 3′-UTR [2572 C > A (rs4846049), 4869 C > G (rs1537514), 5488 C > T (rs3737967), and 6685 T > C (rs4846048)] to elucidate associations between ischemic stroke prevalence and prognosis. We examined 511 consecutive patients with ischemic stroke. Additionally, we selected 411 sex-/age-matched control subjects from patients presenting at our hospitals during the same period. The *MTHFR* 2572 C > A and 6685 T > C were significantly associated with ischemic stroke prevalence in the cardioembolism subgroup (*MTHFR* 2572CC vs. CA + AA: AOR, 2.145; 95% CI, 1.203–3.827; *P* = 0.010; *MTHFR* 6685TT vs. CC: AOR, 10.146; 95% CI, 1.297–79.336; *P* = 0.027). The gene-environment combined effect was significant, with *MTHFR* 2572CA + AA and folate levels ≤3.45 ng/mL correlating with ischemic stroke incidence. In addition, the total homocysteine (tHcy) levels in subjects with *MTHFR* 2572AA were elevated compared to tHcy levels in subjects with *MTHFR* 2572CC. Therefore, we suggest that *MTHFR* 2572 C > A and 6685 T > C are associated with ischemic stroke pathogenesis. The combined effects of the *MTHFR* 3′-UTR polymorphisms and tHcy/folate levels may contribute to stroke prevalence.

## Introduction

Stroke is the third most common cause of death in many developed countries, and approximately 80% of stroke cases are ischemic in origin^1,2^. In South Korea, stroke is the most frequent cause of death after cancer and is more frequent than heart disease^3,4^. Stroke is a complex multifactorial and polygenic disease arising from a variety of gene-gene and gene-environment interactions^5,6^. Multiple factors, including hypertension (HTN), diabetes mellitus (DM), smoking, hyperlipidemia, and hyperhomocysteinemia, are associated with a higher risk of stroke^1,7,8^. In particular, hyperhomocysteinemia is considered an independent, potentially modifiable risk factor for ischemic stroke, and has been previously reported in several studies involving different ethnic groups^9–12^.

The 5, 10-methylenetetrahydrofolate reductase (MTHFR) protein performs a central reaction in folate metabolism. It irreversibly catalyzes the conversion of 5, 10-methylenetetrahydrofolate to 5-methyltetrahydrofolate, the primary circulating form of folate^13,14^. In addition, MTHFR converts 5, 10-methylenetetrahydrofolate into 5-methyltetrahydrofolate and provides the methyl group for homocysteine (Hcy) in methionine synthesis. It has been reported that elevation of Hcy in the blood is associated with an increased risk for arteriosclerosis, myocardial infarction, venous thrombosis, stroke, and neural tube defects^3,15^. The effect of several polymorphic genes involved in folate metabolism, including *MTHFR* on ischemic stroke susceptibility and progression, has been reported. The *MTHFR* gene is critical for Hcy and folate metabolism, and polymorphic variants of the enzymes involved in Hcy and folate metabolism also play an important role in determining the susceptibility of an individual to disease^16^. Lower MTHFR enzyme activity, which can increase total plasma homocysteine (tHcy) levels and decrease plasma folate levels, contributes to stroke development^16–18^. Folate concentrations inversely correlate with tHcy levels^19^. The role of hyperhomocysteinemia in vascular and thromboembolic disease has been extensively studied. Previous studies reported significant vascular disease in patients with markedly elevated tHcy levels^20–22^. The tHcy is hypothesized to increase thrombotic risk by inducing endothelial injury in venous and arterial vasculatures^21^. Abnormal folate concentrations have also been implicated in the development of diseases, such as cardiovascular diseases, neural tube defects, cleft lip and palate, late pregnancy complications, and neurodegenerative and psychiatric disorders^23,24^. Recent studies have shown the clinical impacts of polymorphisms in the 3′-UTR of certain genes, which may potentially bind to specific microRNAs (miRNAs) in various diseases^25–27^. However, variants in the *MTHFR* 3′-UTR have not been extensively examined.

In the present study, we selected four single nucleotide polymorphisms (SNPs) in the *MTHFR* 3′-UTR region: *MTHFR* 2572 C > A (rs4846049), 4869 C > G (rs1537514), 5488 C > T (rs3737967), and 6685 T > C (rs4846048). We then determined their associations with ischemic stroke prevalence and prognosis. The minor allele frequencies of the studied polymorphisms are higher than 5% in the Asian population, and little is known about their genetic associations with ischemic stroke. Therefore, we investigated whether *MTHFR* 3′-UTR polymorphisms correlate with ischemic stroke susceptibility in Korean subjects.

## Results

### Genetic susceptibility of single markers in ischemic stroke subtypes and control subjects

We investigated the *MTHFR* 2572 C > A, 4869 C > G, 5488 C > T, and 6685 T > C polymorphisms. Table 1 shows their genotype distributions in ischemic stroke patients and control subjects. The *MTHFR* 3′-UTR polymorphisms were not significantly correlated with ischemic stroke prevalence. To examine whether the effect of each polymorphism was confined to a specific subtype or related to generalized risks, we further separated the stroke group into three subgroups [large arterial disease (LAD), small-vessel disease (SVD), and, cardioembolism (CE)] according to the Trial of Org 10172 in Acute Stroke Treatment (TOAST) classification^25^. In subgroup analyses, LAD was significantly associated with the *MTHFR* 6685 T > C polymorphism [TT vs. TC: adjusted odds ratio (AOR), 0.517; 95% confidence interval (CI), 0.308–0.867; *P* = 0.012; TT vs. TC + CC: AOR, 0.543; 95% CI, 0.328–0.899; *P* = 0.018]. The *MTHFR* 2572 C > A polymorphism (CC vs. CA: AOR, 2.098; 95% CI, 1.160–3.793; *P* = 0.014; CC vs. CA + AA: AOR, 2.145; 95% CI, 1.203–3.827; *P* = 0.010) and 6685 T > C polymorphism (TT vs. CC: AOR, 10.146; 95% CI, 1.297–79.336; *P* = 0.027; TT + TC vs. CC: AOR, 10.504; 95% CI, 1.373–80.377; *P* = 0.024) were significantly associated with the CE type (Table 1). In addition, we analyzed the association with/without patients of undetermined subtypes for ischemic stroke susceptibilities (Supplemental Table 1).

**Table 1:** Comparison of genotype frequencies and AOR of *MTHFR* gene polymorphisms between the ischemic stroke, subtypes, and control subjects

Genotypes	Controls (n = 411)	Case (n = 511)	AOR (95% CI)*	*P* ^*†*^	LAD (n = 205)	AOR (95% CI)*	*P* ^*†*^	SVD (n = 149)	AOR (95% CI)*	*P* ^*†*^	CE (n = 55)	AOR (95% CI)*	*P* ^*†*^
***MTHFR*** **2572** **C** **>** **A**													
CC	280 (68.1)	333 (65.2)	1.000 (reference)		137 (66.8)	1.000 (reference)		97 (65.1)	1.000 (reference)		28 (50.9)	1.000 (reference)	
CA	122 (29.7)	163 (31.9)	1.216 (0.90–1.638)	0.199	64 (31.2)	1.126 (0.767–1.653)	0.544	47 (31.5)	1.222 (0.794–1.880)	0.363	25 (45.5)	**2.098 (1.160**–**3.793)**	**0.014**
AA	9 (2.2)	15 (2.9)	1.575 (0.653–3.800)	0.312	4 (2.0)	0.932 (0.262–3.315)	0.913	5 (3.4)	1.718 (0.528–5.586)	0.369	2 (3.6)	2.432 (0.463–12.767)	0.294
Dominant (CC vs. CA + AA)			1.242 (0.929–1.660)	0.143		1.120 (0.769–1.631)	0.556		1.266 (0.834–1.924)	0.268		**2.145 (1.203**–**3.827)**	**0.010**
Recessive (CC + CA vs. AA)			1.499 (0.624–3.600)	0.365		0.966 (0.275–3.391)	0.957		1.645 (0.516–5.239)	0.400		2.027 (0.408–10.072)	0.388
HWE-*P*	0.308	0.352											
***MTHFR*** **4869 C > G**													
CC	368 (89.5)	457 (89.4)	1.000 (reference)		179 (87.3)	1.000 (reference)		134 (89.9)	1.000 (reference)		50 (90.9)	1.000 (reference)	
CG	43 (10.5)	54 (10.6)	1.075 (0.689–1.676)	0.750	26 (12.7)	1.379 (0.797–2.388)	0.251	15 (10.1)	0.980 (0.503–1.909)	0.952	5 (9.1)	0.822 (0.304–2.220)	0.699
GG	0 (0.0)	0 (0.0)	N/A		0 (0.0)	N/A		0 (0.0)	N/A		0 (0.0)	N/A	
Dominant (CC vs. CG + GG)			1.075 (0.689–1.676)	0.750		1.379 (0.797–2.388)	0.251		0.980 (0.503–1.909)	0.952		0.822 (0.304–2.220)	0.699
Recessive (CC + CG vs. GG)			N/A			N/A			N/A			N/A	
HWE-*P*	0.263	0.208											
***MTHFR*** **5488 C > T**													
CC	351 (85.4)	450 (88.1)	1.000 (reference)		177 (86.3)	1.000 (reference)		131 (87.9)	1.000 (reference)		49 (89.1)	1.000 (reference)	
CT	59 (14.4)	57 (11.2)	0.837 (0.556–1.258)	0.391	28 (13.7)	1.134 (0.681–1.890)	0.629	16 (10.7)	0.740 (0.396–1.385)	0.347	6 (10.9)	0.754 (0.304–1.871)	0.543
TT	1 (0.2)	4 (0.8)	2.738 (0.296–25.290)	0.375	0 (0.0)	N/A	0.998	2 (1.3)	4.486 (0.391–51.468)	0.228	0 (0.0)	N/A	0.998
Dominant (CC vs. CT + TT)			0.875 (0.586–1.306)	0.513		1.120 (0.673–1.864)	0.664		0.824 (0.452–1.501)	0.526		0.748 (0.301–1.855)	0.531
Recessive (CC + CT vs. TT)			2.857 (0.307–26.552)	0.356		N/A	0.998		4.679 (0.402–54.450)	0.218		N/A	0.998
HWE-*P*	0.364	0.151											
***MTHFR*** **6685 T > C**													
TT	326 (79.3)	426 (83.4)	1.000 (reference)		180 (87.8)	1.000 (reference)		122 (81.9)	1.000 (reference)		40 (72.7)	1.000 (reference)	
TC	83 (20.2)	78 (15.3)	0.795 (0.556–1.137)	0.209	23 (11.2)	**0.517 (0.308**–**0.867)**	**0.012**	25 (16.8)	0.921 (0.551–1.540)	0.753	13 (23.6)	1.297 (0.655–2.567)	0.456
CC	2 (0.5)	7 (1.4)	2.902 (0.553–15.240)	0.208	2 (1.0)	1.418 (0.174–11.537)	0.744	2 (1.3)	2.775 (0.326–23.649)	0.351	2 (3.6)	**10.146 (1.297**–**79.336)**	**0.027**
Dominant (TT vs. TC + CC)			0.846 (0.597–1.200)	0.349		**0.543 (0.328**–**0.899)**	**0.018**		0.966 (0.585–1.595)	0.892		1.481 (0.773–2.837)	0.237
Recessive (TT + TC vs. CC)			3.199 (0.613–16.682)	0.168		1.693 (0.209–13.693)	0.622		2.842 (0.349–23.145)	0.329		**10.504 (1.373**–**80.377)**	**0.024**
HWE-*P*	0.175	0.123											

Abbreviation; AOR, adjusted odds ratio; MTHFR, methylenetetrahydrofolate reductase; CI, confidence interval; LAD, large artery disease; SVD, small vessel disease; CE, cardioembolism; HWE, Hardy-Weinberg equilibrium.

^†^The *P*-value calculated by multiple logistic regression on the basis of risk factors such as age, gender, hypertension, hyperlipidemia, and diabetes mellitus.

### Combined effects of *MTHFR* gene polymorphisms and clinical parameters on disease prevalence

To determine additional clinical significance, we evaluated the combined effects of the gene environment. The *MTHFR* gene polymorphism elevated stroke prevalence in several conditions, including hypertension, diabetes mellitus, hyperlipidemia, smoking, high density lipoprotein-cholesterol levels, triglyceride levels, folate levels ≤3.45 nmol/mL, and tHcy levels ≥11.22 μmol/L (Table 2). The *MTHFR* 2572 CA + AA genotype was shown to have synergic effects with ischemic stroke prevalence. In particular, low folate levels were the most predictive, with *MTHFR* 2572CA + AA and folate ≤3.45 nmol/L (AOR, 6.532; 95% CI, 2.592–16.46) shown to significantly increase ischemic stroke incidence. In addition, the *MTHFR* 4869CG + GG genotype had a combinatorial effect with hypertension (AOR, 3.217; 95% CI, 1.763–5.872) and smoking (AOR, 5.067; 95% CI, 1.788–14.360), whereas the *MTHFR* 5488CT + TT genotype was significant only in the smoking group (AOR, 2.740; 95% CI, 1.196–6.278). In addition, we performed stratified analyses for clinical factors including sex, age, hypertension, diabetes mellitus, hyperlipidemia, smoking status, folate levels, and homocysteine levels (Supplemental Table 2).

**Table 2:** Ischemic stroke incidence by interactions with hypertension, diabetes mellitus, hyperlipidemia, smoking, HDL-cholesterol, folate, and homocysteine.

Characteristics	*MTHFR* 2572 C > A		*MTHFR* 4869 C > G		*MTHFR* 5488 C > T		*MTHFR* 6685 T > C	
	CC	CA + AA	CC	CG + GG	CC	CT + TT	TT	TC + CC
Hypertension								
No	1.000 (reference)	1.020 (0.667–1.558)	1.000 (reference)	1.095 (0.569–2.106)	1.000 (reference)	0.739 (0.412–1.327)	1.000 (reference)	0.762 (0.465–1.250)
Yes	0.834 (0.484–1.439)	**3.525 (2.282–5.447)**	2.469 (1.840–3.313)	2.952 (1.572–5.546)	2.363 (1.754–3.185)	2.809 (1.543–5.116)	2.409 (1.766–3.285)	2.211 (1.302–3.753)
Diabetes Mellitus								
No	1.000 (reference)	1.398 (1.016–1.924)	1.000 (reference)	1.123 (0.693–1.818)	1.000 (reference)	0.923 (0.597–1.426)	1.000 (reference)	0.956 (0.651–1.403)
Yes	0.906 (0.526–1.561)	**1.921 (1.053–3.504)**	2.222 (1.529–3.231)	1.824 (0.608–5.473)	2.236 (1.529–3.270)	1.459 (0.553–3.849)	2.467 (1.653–3.683)	1.174 (0.549–2.511)
Hyperlipidemia								
No	1.000 (reference)	1.263 (0.903–1.766)	1.000 (reference)	1.149 (0.696–1.897)	1.000 (reference)	0.968 (0.613–1.529)	1.000 (reference)	0.885 (0.589–1.329)
Yes	0.873 (0.503–1.518)	1.557 (0.934–2.594)	1.362 (0.979–1.896)	1.050 (0.421–2.620)	1.392 (0.993–1.950)	0.832 (0.373–1.852)	1.379 (0.969–1.962)	1.025 (0.548–1.918)
Smoking								
No	1.000 (reference)	1.069 (0.748–1.527)	1.000 (reference)	0.750 (0.449–1.255)	1.000 (reference)	0.670 (0.418–1.073)	1.000 (reference)	0.846 (0.547–1.309)
Yes	0.828 (0.477–1.437)	**1.979 (1.142–3.430)**	1.159 (0.818–1.641)	**4.929 (1.697–14.322)**	1.133 (0.797–1.611)	**2.748 (1.167–6.473)**	1.239 (0.854–1.797)	1.200 (0.644–2.235)
HDL-Cholesterol*								
≥40/50 mg/dl	1.000 (reference)	1.095 (0.770–1.557)	1.000 (reference)	1.304 (0.769–2.212)	1.000 (reference)	1.050 (0.647–1.706)	1.000 (reference)	0.583 (0.378–0.901)
<40/50 mg/dl	0.862 (0.496–1.501)	**3.011 (1.817–4.990)**	2.192 (1.578–3.045)	1.646 (0.717–3.779)	2.206 (1.579–3.082)	1.382 (0.678–2.817)	1.586 (1.130–2.228)	**3.226 (1.632**–**6.380)**
Triglyceride								
≤150 mg/dl	1.000 (reference)	1.369 (0.957–1.958)	1.000 (reference)	0.772 (0.460–1.298)	1.000 (reference)	0.721 (0.443–1.172)	1.000 (reference)	1.154 (0.745–1.786)
>150 mg/dl	0.902 (0.518–1.573)	1.376 (0.839–2.257)	0.907 (0.655–1.254)	**2.590 (1.034–6.490)**	0.930 (0.670–1.291)	1.343 (0.656–2.750)	1.189 (0.848–1.667)	0.609 (0.335–1.108)
Folate^†^								
>3.45 nmol/L	1.000 (reference)	1.280 (0.936–1.750)	1.000 (reference)	1.105 (0.689–1.772)	1.000 (reference)	0.883 (0.574–1.359)	1.000 (reference)	0.887 (0.609–1.294)
≤3.45 nmol/L	0.902 (0.518–1.573)	**6.532 (2.592**–**16.458)**	3.929 (2.448–6.307)	4.821 (0.971–23.941)	3.825 (2.369–6.176)	**4.197 (1.099–16.029)**	3.764 (2.292–6.180)	3.272 (1.124–9.523)
Homocysteine^‡^								
<11.22 μmol/L	1.000 (reference)	1.230 (0.900–1.680)	1.000 (reference)	1.008 (0.626–1.625)	1.000 (reference)	0.825 (0.537–1.265)	1.000 (reference)	0.907 (0.624–1.319)
≥11.22 μmol/L	0.862 (0.496–1.501)	**2.733 (1.284–5.817)**	1.576 (1.035–2.399)	2.850 (0.744–10.919)	1.550 (1.017–2.362)	2.805 (0.734–10.719)	1.757 (1.125–2.745)	1.118 (0.442–2.828)

Adjusted by age, sex, hypertension, diabetes mellitus, hyperlipidemia, and smoking. Abbreviations are defined in Table 1.

*HDL-cholesterol has a different standard value for males and females, with 40 for males and 50 for females.

^†^3.16 nmol/L is based on the bottom 15% of folate levels in patients and controls.

^‡^16.36 μmol/L is based on the top 15% of the homocysteine levels in patients and controls.

### Haplotype analysis with disease incidence

The linkage disequilibrium (LD) of *MTHFR* polymorphisms at loci 2572 (rs4846049)/4869, (rs1537514)/5488, (rs3737967)/6685 (rs4846048) in ischemic stroke patients and control subjects is shown in the Supplemental Figure 1. There was a strong LD between loci 4869/5488 (D’ = 0.944), 2572/5488 (D’ = 0.949), and 2572/4869 (D’ = 0.938). To evaluate the combined effects of the *MTHFR* 3′-UTR SNP loci on ischemic stroke incidence, logistic regression for the combined genotypes and haplotypes was performed. The 2572CC/4869CC/5488CC/6685TC (AOR, 0.095; 95% CI, 0.012–0.761; *P* = 0.027), 2572CA/4869CC/5488CC/6685TT (AOR, 2.922, 95% CI, 1.342–6.361; *P* = 0.007), 2572A-4869C-5488C-6685T (AOR, 0.062; 95% CI, 0.008–0.477; *P* = 0.001), and 2572A-4869C-5488C-6685T (AOR, 2.239, 95% CI, 1.178–4.257, *P* = 0.013) types contributed to ischemic stroke prevalence (Table 3). In the ischemic stroke subgroup, the *MTHFR* haplotypes were shown to have a significant association with CE subtype prevalence (Supplemental Table 3).

**Table 3:** Combined genotype and allele frequencies of the *MTHFR* 3′-UTR polymorphisms for ischemic stroke patients and control subjects.

Genotypes	Controls freq. (n = 411)	Case freq. (n = 511)	AOR (95% CI)	*P* ^†^	*P* ^‡^
*MTHFR* 2572/4869/5488/6685 genotype combination					
CC/CC/CC/TT	0.652	0.644	1.000 (reference)		
**CC/CC/CC/TC**	**0.029**	**0.002**	**0.095 (0.012–0.761)**	**0.027**	0.149
CC/CC/CT/TT	0.000	0.002	0.393 (0.021–7.323)	0.531	0.680
**CA/CC/CC/TT**	**0.022**	**0.065**	**2.922 (1.342–6.361)**	**0.007**	0.077
CA/CC/CC/TC	0.141	0.145	1.106 (0.745–1.642)	0.618	0.680
CA/CC/CT/TT	0.024	0.010	0.475 (0.157–1.438)	0.188	0.528
CA/CC/CT/TC	0.010	0.002	0.309 (0.034–2.844)	0.299	0.581
CA/CG/CT/TT	0.085	0.092	1.218 (0.746–1.989)	0.431	0.677
AA/CC/CC/TT	0.005	0.002	0.524 (0.044–6.192)	0.608	0.680
AA/CC/CC/CC	0.005	0.014	3.004 (0.575–15.704)	0.192	0.528
AA/CC/CT/TC	0.005	0.002	0.628 (0.056–7.100)	0.707	0.707
AA/CG/CT/TC	0.007	0.002	0.295 (0.027–3.213)	0.317	0.581
*MTHFR* 2572/4869/5488/6685 allele combination					
C-C-C-T	0.812	0.807	1.000 (reference)		
**C-C-C-C**	**0.016**	**0.001**	**0.062 (0.008–0.477)**	**0.001**	0.006
C-C-T-T	0.001	0.001	0.809 (0.050–12.96)	1.000	1.000
C-G-C-T	0.000	0.002	4.043 (0.194–84.42)	0.505	0.758
C-G-T-T	0.001	0.000	0.270 (0.011–6.632)	0.447	0.758
C-G-T-C	0.001	0.000	0.270 (0.011–6.632)	0.447	0.758
**A-C-C-T**	**0.016**	**0.035**	**2.239 (1.178–4.257)**	**0.013**	0.078
A-C-C-C	0.081	0.088	1.086 (0.779–1.514)	0.673	0.897
A-C-T-T	0.016	0.014	0.871 (0.406–1.865)	0.846	1.000
A-C-T-C	0.005	0.001	0.202 (0.023–1.814)	0.180	0.600
A-G-C-T	0.000	0.003	5.660 (0.292–109.9)	0.258	0.619
A-G-T-T	0.047	0.048	1.016 (0.659–1.566)	1.000	1.000
A-G-T-C	0.003	0.000	0.162 (0.008–3.377)	0.200	0.600

^†^The *P*-value was calculated by multiple logistic regression on the basis of risk factors such as age, gender, hypertension, hyperlipidemia, and diabetes mellitus. Abbreviations are defined in Table 1.

^‡^The false discovery rate-adjusted *P* value for multiple hypothesis testing using the Benjamini-Hochberg method.

### Differences of blood coagulation factors according to *MTHFR* polymorphisms

Analyses of variance were used to show differences in blood coagulant factors (fibrinogen, antithrombin, platelet, activated partial thromboplastin time, and prothrombin time), folate, and tHcy levels according to genotype (Table 4). The tHcy levels in subjects with the *MTHFR* 2572AA polymorphism were elevated compared to the tHcy levels in subjects with the *MTHFR* 2572CC (*P* = 0.011). Additionally, the *MTHFR* 5488 and 6685 mutant genotypes had higher tHcy levels than that of the wild-type genotypes (*MTHFR* 5488, *P* = 0.020; *MTHFR* 6685, *P* = 0.005). Supplemental Table 4 shows the combination models with *MTHFR* 677 C > T, which measured the Hcy levels. The *MTHFR* 677-2572 combination group (677CC-2572AA: 12.14 ± 5.56; *P* = 0.010) and *MTHFR* 677-6685 combination group (677CC-6685CC: 14.61 ± 6.53; *P* < 0.0001) were significantly different compare to each wild type genotype.

**Table 4:** Altered plasma folate and homocysteine levels, and blood coagulation factors according to *MTHFR* genotypes.

Genotypes	Folate (nmol/L)		tHcy (μmol/L)		Fibrinogen (mg/dL)		Antithrombin (%)		PLT (10^3^cell/μL)		aPTT (sec)		PT (sec)	
	Mean ± SD (N)	CV, %	Mean ± SD (N)	CV, %	Mean ± SD (N)	CV, %	Mean ± SD (N)	CV, %	Mean ± SD (N)	CV, %	Mean ± SD (N)	CV, %	Mean ± SD (N)	CV, %
*MTHFR* 2572 C > A														
CC	7.56 ± 5.60 (608)	74.1	10.89 ± 5.33 (612)	48.9	418.07 ± 128.28 (407)	30.7	94.80 ± 29.46 (408)	31.1	246.33 ± 76.57 (612)	31.1	30.89 ± 6.47 (536)	20.9	11.75 ± 0.80 (536)	6.8
CA	8.05 ± 5.93 (281)	73.7	9.94 ± 3.77 (281)	37.9	420.94 ± 126.74 (190)	30.1	93.34 ± 16.82 (191)	18.0	239.85 ± 64.13 (279)	26.7	32.95 ± 19.32 (237)	58.6	11.85 ± 1.19 (237)	10.0
AA	6.76 ± 4.00 (24)	59.2	11.98 ± 5.50 (24)	45.9	433.22 ± 154.08 (18)	35.6	91.71 ± 17.60 (17)	19.2	265.13 ± 211.05 (23)	79.6	34.11 ± 6.02 (19)	17.6	11.60 ± 0.83 (19)	7.2
*P* ^a^	0.358		0.011*		0.870		0.746		0.242*		0.055*		0.240	
C allele	7.65 ± 5.66 (1497)	74.0	10.71 ± 5.09 (1505)	47.5	418.61 ± 127.87 (1004)	30.5	94.53 ± 27.50 (1007)	29.1	245.13 ± 74.42 (1503)	30.4	31.27 ± 10.11 (1309)	32.3	11.77 ± 0.88 (1309)	7.5
A allele	7.86 ± 5.70 (329)	72.5	10.23 ± 4.11 (329)	40.2	422.89 ± 130.77 (226)	30.9	93.09 ± 16.87 (225)	18.1	243.43 ± 98.26 (325)	40.4	33.11 ± 18.06 (275)	54.5	11.82 ± 1.14 (275)	9.6
*P* ^b^	0.551		0.114		0.651		0.453		0.725		0.019*		0.410	
*MTHFR* 4869 C > G														
CC	7.61 ± 5.68 (817)	74.6	10.74 ± 5.02 (820)	46.7	419.40 ± 129.53 (556)	30.9	94.17 ± 26.70 (556)	28.4	244.94 ± 81.46 (818)	33.3	31.54 ± 12.11 (713)	38.4	11.77 ± 0.95 (712)	8.1
CG	8.36 ± 5.57 (96)	66.6	9.64 ± 4.00 (97)	41.5	419.34 ± 118.47 (59)	28.3	95.12 ± 16.82 (60)	17.7	243.85 ± 56.44 (96)	23.1	32.03 ± 9.79 (79)	30.6	11.85 ± 0.84 (80)	7.1
GG	—		—		—		—		—		—		—	
*P* ^a^	0.222		**0.037**		0.997		0.789		0.899		0.729		0.469	
C allele	7.65 ± 5.67 (1730)	74.1	10.68 ± 4.97 (1737)	46.5	419.40 ± 128.89 (1171)	30.7	94.22 ± 26.27 (1172)	27.9	244.88 ± 80.24 (1732)	32.8	31.56 ± 11.99 (1505)	38.0	11.77 ± 0.94 (1504)	8.0
G allele	8.36 ± 5.57 (96)	66.6	9.64 ± 4.00 (97)	41.5	419.34 ± 118.47 (59)	28.3	95.12 ± 16.82 (60)	17.7	243.85 ± 56.44 (96)	23.1	32.03 ± 9.79 (79)	30.6	11.85 ± 0.84 (80)	7.1
*P* ^b^	0.222		**0.037**		0.997		0.789		0.899		0.729		0.469	
*MTHFR* 5488 C > T														
CC	7.55 ± 5.70 (793)	75.5	10.79 ± 5.06 (796)	46.9	419.00 ± 129.14 (544)	30.8	94.24 ± 26.90 (544)	28.5	245.13 ± 81.59 (796)	33.3	31.43 ± 12.14 (697)	38.6	11.77 ± 0.95 (697)	8.1
CT	8.67 ± 5.43 (115)	62.6	9.45 ± 3.76 (116)	39.8	420.46 ± 115.42 (68)	27.5	94.25 ± 16.62 (70)	17.6	245.49 ± 60.45 (113)	24.6	32.63 ± 9.97 (92)	30.6	11.79 ± 0.79 (92)	6.7
TT	7.04 ± 4.56 (5)	64.8	12.04 ± 4.74 (5)	39.4	466.67 ± 291.23 (3)	62.4	101.50 ± 20.51 (2)	20.2	182.40 ± 25.51 (5)	14.0	35.22 ± 4.02 (3)	11.4	12.08 ± 1.11 (3)	9.2
*P* ^a^	0.139		**0.020**		0.813*		0.925		0.209		0.575		0.845	
C allele	7.63 ± 5.69 (1701)	74.6	10.69 ± 4.99 (1708)	46.7	419.09 ± 128.28 (1156)	30.6	94.24 ± 26.37 (1158)	28.0	245.15 ± 80.32 (1705)	32.8	31.51 ± 12.02 (1486)	38.1	11.77 ± 0.94 (1486)	8.0
T allele	8.54 ± 5.35 (125)	62.6	9.66 ± 3.87 (126)	40.1	424.21 ± 130.52 (74)	30.8	94.65 ± 16.59 (74)	17.5	240.36 ± 60.80 (123)	25.3	32.79 ± 9.71 (98)	29.6	11.80 ± 0.80 (98)	6.8
*P* ^b^	0.084		**0.023**		0.740		0.896		0.517		0.300		0.762	
*MTHFR* 6685 T > C														
TT	7.69 ± 5.55 (743)	72.2	10.71 ± 5.15 (749)	48.1	419.45 ± 129.33 (506)	30.8	95.05 ± 27.38 (508)	28.8	246.20 ± 74.39 (747)	30.2	31.23 ± 7.32 (654)	23.4	11.76 ± 0.80 (655)	6.8
TC	7.73 ± 6.28 (161)	81.2	9.95 ± 3.34 (159)	33.6	415.54 ± 123.05 (102)	29.6	90.04 ± 17.18 (101)	19.1	233.80 ± 62.84 (158)	26.9	33.25 ± 24.31 (130)	73.1	11.86 ± 1.44 (129)	12.1
CC	6.81 ± 4.64 (9)	68.1	15.11 ± 6.72 (9)	44.5	472.14 ± 146.62 (7)	31.1	98.14 ± 11.17 (7)	11.4	325.00 ± 334.80 (9)	103.0	33.41 ± 4.58 (8)	13.7	11.51 ± 0.79 (8)	6.9
*P* ^a^	0.895		0.005*		0.530		0.191		0.002*		0.192*		0.389	
T allele	7.70 ± 5.62 (1647)	73.0	10.64 ± 5.01 (1657)	47.1	419.09 ± 128.66 (1114)	30.7	94.60 ± 26.64 (1117)	28.2	245.01 ± 73.42 (1652)	30.0	31.42 ± 10.11 (1438)	32.2	11.77 ± 0.88 (1439)	7.5
C allele	7.63 ± 6.12 (179)	80.2	10.47 ± 4.07 (177)	38.9	422.37 ± 126.03 (116)	29.8	91.03 ± 16.71 (115)	18.4	243.13 ± 120.66 (176)	49.6	33.27 ± 22.98 (146)	69.1	11.82 ± 1.38 (145)	11.7
*P* ^b^	0.886		0.665		0.793		0.159		0.764		0.073*		0.530	

Abbreviations: tHcy, total plasma homocysteine; PLT, platelet; aPTT, activated partial thromboplastin time; PT, prothrombin time. Other abbreviations are defined in Table 1.

^a^One-way analysis of variance test. ^b^ Independent two-sample *t*-test.

### Survival analysis of *MTHFR* polymorphisms and ischemic stroke mortality

During a mean follow-up period of 7 years, 98 patients (17.5%) died. We investigated whether the *MTHFR* 3′-UTR genotypes were associated with long-term overall survival (OS) after ischemic stroke using Kaplan-Meier analyses. However, we did not find significant associations with individual *MTHFR* 3′-UTR genotypes (Supplemental Figure 2).

## Discussion

Hcy is a well-known thrombotic factor in vascular diseases including coronary artery disease^28^, heart disease^29^, arteriosclerosis^3,15^, myocardial infarction^30^, venous thrombosis^31^, chronic kidney disease^32^, and ischemic stroke^33–35^. There is increasing evidence that Hcy may affect the coagulation system and the resistance of the endothelium to thrombosis^36^. Moreover, Hcy may interfere with the vasodilator and antithrombotic functions of nitric oxide^37^. Notably, vascular complications reported in patients with homocystinuria are related to thrombosis rather than atherosclerosis^38^, and a relationship between tHcy levels and the incidence of thrombotic events has recently been reported in patients with systemic lupus erythematosus^30^.

Previous studies have identified the *MTHFR* gene as being associated with ischemic stroke prevalence^33–37^. Numerous studies reported that *MTHFR* 677 C > T was associated with increased stroke risk^34,35^, likely because the *MTHFR* 677 T allele decreased *MTHFR* gene activity^35^. Other studies reported that the methylation pattern of CpG island regions in the *MTHFR* gene had decreased MTHFR activity, causing an abnormality for tHcy and serum folate levels that were associated with ischemic stroke occurrence^36,37^. Moreover, we have shown an association with ischemic stroke risk based on computational epigenetic profiling of CpG islands in the *MTHFR* gene^37^. However, epigenetic regulation of the *MTHFR* gene could occur via another mechanism that included RNA interference with miRNA binding. In addition, a previous study showed differential mRNA expression levels of the *MTHFR* gene according to 3′-UTR polymorphisms^38,39^.

Therefore, understanding the genesis of ischemic events due to decreased *MTHFR* activity might explain why 3′-UTR polymorphisms could affect ischemic events, stroke occurrence, and patient prognosis. Polymorphisms in the 3′-UTR region could affect mRNA stability and translation, which may significantly impact gene expression by abolishing, weakening, or creating miRNA binding sites. Currently, there are not sufficient data to indicate that miRNA binding activity is modulated depending on *MTHFR* 3′-UTR polymorphisms. One study reported that miR-149 binding activity was affected by the *MTHFR* 2572 C > A polymorphism in coronary heart disease risk^38^. Therefore, we investigated *MTHFR* 3′-UTR polymorphisms that have potential miRNA binding sites. We found that these regions were capable of binding with miRNA. Despite the lack of data, these miRNAs may be important genetic factors for the prevalence and progression of ischemic stroke because their expression is altered in some genotypes^38^.

In conclusion, we investigated the relationship of the *MTHFR* 2572 C > A and 6685 T > C polymorphisms with ischemic stroke incidence and progression. The *MTHFR* 2572 C > A and 6685 T > C polymorphisms were shown to increase the risk of embolisms of cardiac origin and ischemic stroke occurrence, and the prevalence of large-artery-origin ischemic stroke had a decreased odds ratio (OR) for the *MTHFR* 6685 T > C polymorphism. Moreover, the combination of *MTHFR* 2572CA + AA genotypes and serum folate levels or tHcy levels were synergic for ischemic stroke susceptibility, whereas other *MTHFR* 3′-UTR polymorphisms were not associated with serum folate and tHcy for ischemic stroke risk. Therefore, we hypothesized that the *MTHFR* 3′-UTR regulates one-carbon metabolism through miRNA binding. We found that these genotypes and haplotypes positively correlated with the occurrence and unfavorable prognosis of stroke, according to vascular disease risk factors, including hypertension, diabetes mellitus, HDL-C levels, tHcy levels, and folate levels.

This study has several limitations. First, the mechanisms by which 3′-UTR polymorphisms in the *MTHFR* gene affect stroke development remain unclear. Second, the controls in our study were not completely healthy because some of them were seeking medical attention. However, the recruitment of only healthy participants for imaging and laboratory tests would markedly reduce the enrollment number, and enrollment of participants without imaging and laboratory tests may produce other biases in vascular risk factor assessment. Third, information regarding additional environmental risk factors in stroke patients remains to be investigated. Finally, the population of this study was restricted to patients of Korean ethnicity. Although the results from this study provide the first evidence for 3′-UTR variants in *MTHFR* as potential biomarkers of stroke prevention and prognosis, a prospective study using a larger cohort of patients is warranted to validate these findings.

## Methods

### Ethics statement

All study protocols of participants were reviewed and approved by The Institutional Review Board of CHA Bundang Medical Center and followed the recommendations of the Declaration of Helsinki. Study subjects were recruited from the South Korean provinces of Seoul and Gyeonggi-do between 2000 and 2008. The Institutional Review Board of CHA Bundang Medical Center approved this genetic study in June 2000 and informed consent was obtained from the study participants.

### Study population

We included 511 consecutive patients with ischemic stroke referred from the Department of Neurology at CHA Bundang Medical Center, CHA University. Ischemic stroke was defined as a stroke (a clinical syndrome characterized by rapidly developing clinical symptoms and signs of focal or global loss of brain function) with evidence of cerebral infarction in clinically relevant areas of the brain according to brain imaging using magnetic resonance imaging (MRI). The date and cause of death were identified using death certificates from the Korean National Statistical Office. Patients who were alive on Dec 31, 2012 were censored at that point. The death statistics of the Korean National Statistical Office have been previously reported to be reliable^40^.

Based on clinical manifestations and neuroimaging data, two neurologists classified all ischemic strokes into four causative subtypes using the TOAST criteria as follows: (1) large-artery disease (LAD), significant (≥50%) stenosis of a relevant cerebral artery confirmed by cerebral angiography; (2) small-vessel disease (SVD), an infarction lesion <15 mm in diameter, and classic lacunar syndrome without evidence of a cerebral cortical dysfunction or potentially detectable cardiac sources for embolism; (3) cardioembolism (CE), presumably due to an embolus arising in the heart, as detected by cardiac evaluation; and (4) undetermined pathogenesis, in which the cause of stroke could not be determined or patients with two or more potential causes^41^. The frequencies of the stroke subtypes were 40% LAD (n = 205), 29% SVD (n = 149), 11% CE (n = 55), and 20% undetermined pathogenesis (n = 102). These proportions are similar to previously reported values for the Korean population^42^.

We selected 411 sex- and age-matched (±5 years) control subjects from patients presenting at our hospitals during the same period for health examinations, including biochemical testing, electrocardiogram analyses, and brain MRI. Control subjects did not have a recent history of cerebrovascular disease or myocardial infarction. Exclusion criteria were the same as those used in the patient group. The demographic and laboratory data of patients with ischemic stroke, subtype patients [LAD, SVD, and CE] and control subjects are summarized in Table 5. In our sample, 43.1% and 42.1% of stroke patients and control subjects were male, respectively. The mean ages of stroke patients and the control population were 62.96 ± 10.90 years and 62.82 ± 10.61 years, respectively. There were few significant differences between the two groups. Ischemic stroke patients were significantly more likely to have metabolic syndrome, as well as DM, hypertension, fibrinogen, increased tHcy levels, and decreased folate levels (*P* < 0.05).

**Table 5:** Baseline characteristics of control and stroke patients

Characteristic	Controls (n = 411)	Stroke patients (n = 511)	*P*	LAD patients (n = 205)	*P*	SVD patients (n = 149)	*P*	CE patients (n = 55)	*P*
Male (%)	173 (42.1)	220 (43.1)	0.853	85 (41.5)	0.924	72 (48.3)	0.416	22 (40.0)	0.849
Age (years, mean ± SD)	62.82 ± 10.61	62.96 ± 10.90	0.841	64.10 ± 10.43	0.156	60.89 ± 10.87	0.059	65.80 ± 11.87	0.054
Smoking (%)	138 (33.6)	194 (38.0)	0.343	79 (38.5)	0.404	58 (38.9)	0.420	17 (30.9)	0.779
MetS (%)	112 (27.3)	204 (39.9)	0.005	87 (42.4)	0.008	64 (43.0)	0.013	14 (25.5)	0.830
BMI (kg/cm^2^, mean ± SD)	24.29 ± 3.22	24.19 ± 3.06	0.666	24.27 ± 2.99	0.936	24.15 ± 3.08	0.678	23.71 ± 3.15	0.227
Hypertension (%)	169 (41.1)	328 (64.2)	0.0001	135 (65.9)	0.001	91 (61.1)	0.014	31 (56.4)	0.193
SBP (mmHg, mean ± SD)	132.12 ± 17.05	138.29 ± 23.11	0.0001*	139.93 ± 24.20	0.0001*	139.48 ± 20.90	0.0001*	133.42 ± 25.44	0.976*
DBP (mmHg, mean ± SD)	80.42 ± 11.53	83.12 ± 12.39	0.0007	84.45 ± 12.46	0.0001	83.18 ± 11.60	0.012	81.18 ± 17.08	0.821*
Diabetes mellitus (%)	54 (13.1)	141 (27.6)	<0.0001	57 (27.8)	0.0003	45 (30.2)	0.0002	10 (18.2)	0.383
FBS (mg/dL, mean ± SD)	113.86 ± 35.62	137.52 ± 59.46	<0.0001*	139.63 ± 62.59	<0.0001*	137.13 ± 55.74	<0.0001*	138.07 ± 61.10	<0.0001*
Hyperlipidemia (%)	95 (23.1)	150 (29.4)	0.104	65 (31.7)	0.082	44 (29.5)	0.234	10 (18.2)	0.507
HDL-C (mg/dl, mean ± SD)	46.42 ± 13.72	44.46 ± 15.57	0.143	43.26 ± 13.09	0.024	44.50 ± 13.61	0.217	46.27 ± 13.93	0.944
LDL-C (mg/dl, mean ± SD)	118.10 ± 42.12	120.96 ± 33.51	0.105*	126.38 ± 38.32	0.049	116.51 ± 29.15	0.826*	115.15 ± 26.96	0.829*
T. chol (mg/dl, mean ± SD)	192.90 ± 37.51	190.84 ± 40.31	0.431	194.45 ± 45.17	0.932*	189.18 ± 37.19	0.303	180.62 ± 34.92	0.022
Triglyceride (mg/dl, mean ± SD)	147.01 ± 90.22	155.34 ± 114.85	0.359*	151.22 ± 97.68	0.599	168.18 ± 124.29	0.101*	134.87 ± 177.75	0.013*
Platelet (10^3^ cell/μL, mean ± SD)	242.23 ± 67.43	246.89 ± 87.38	0.750*	256.16 ± 88.02	0.352*	236.71 ± 63.26	0.385	244.71 ± 145.70	0.069*
Prothrombin time (sec, mean ± SD)	11.77 ± 0.80	11.78 ± 1.01	0.598*	11.77 ± 0.75	0.992	11.64 ± 0.79	0.109	12.00 ± 1.02	0.168*
aPTT (sec, mean ± SD)	33.43 ± 18.50	30.50 ± 4.48	0.042*	30.39 ± 4.67	0.022*	30.85 ± 4.65	0.097*	30.74 ± 4.31	0.685*
Antithrombin (%, mean ± SD)	94.64 ± 43.45	94.15 ± 17.39	0.129*	95.36 ± 15.51	0.072*	95.21 ± 19.77	0.099*	88.09 ± 17.05	0.168*
Fibrinogen (mg/dL, mean ± SD)	398.18 ± 120.27	425.94 ± 130.25	0.023	433.71 ± 132.31	0.012	396.43 ± 111.67	0.899	447.77 ± 134.16	0.014
D-dimer (ng/mL, mean ± SD)	902.69 ± 1562.75	812.42 ± 1674.29	0.580	853.93 ± 2368.48	0.839	653.71 ± 996.72	0.138	1001.09 ± 947.66	0.0003*
tHcy (μmol/L, mean ± SD)	10.06 ± 4.19	11.07 ± 5.41	0.001*	11.22 ± 6.03	0.027*	10.96 ± 5.01	0.023*	10.01 ± 4.72	0.928
Folate (nmol/L, mean ± SD)	8.67 ± 6.25	6.90 ± 5.03	<0.0001*	6.55 ± 4.32	<0.0001*	7.10 ± 5.69	0.007	7.60 ± 5.37	0.228
Creatinine (mg/dL, mean ± SD)	0.96 ± 0.25	1.01 ± 0.63	0.849	1.03 ± 0.72	0.825*	0.99 ± 0.64	0.344*	0.95 ± 0.34	0.231*
BUN (mg/dL, mean ± SD)	15.84 ± 5.01	15.96 ± 6.27	0.630*	15.40 ± 4.94	0.303	15.11 ± 5.21	0.133	18.94 ± 10.87	0.029*
Vitamin B12 (pg/mL, mean ± SD)	746.55 ± 665.74	748.05 ± 647.22	0.973	816.00 ± 904.59	0.934*	658.09 ± 309.93	0.140*	743.24 ± 286.73	0.154*

Abbreviations: MetS, metabolic syndrome; BMI, body mass index; SBP, systolic blood pressure; DBP, diastolic blood pressure; FBS, fasting blood sugar; HDL-C, high density lipoprotein cholesterol; LDL-C, low density lipoprotein cholesterol; T. chol, total cholesterol; aPTT, activated partial thromboplastin time; tHcy, total plasma homocysteine; BUN, blood urea nitrogen. Other abbreviations are defined in Table 1.

*P-values* were calculated by two-sided t-test for continuous variables and chi-square test for categorical variables.

### Estimation of tHcy and folate levels

Within 48 hours of stroke onset, we collected plasma samples to measure tHcy and folate levels. Twelve hours after the patient’s previous meal, we collected whole blood in a tube containing anticoagulant. Tubes were centrifuged for 15 minutes at 1000 × *g* to separate the plasma. The tHcy concentrations were measured using a fluorescent polarizing immunoassay with the IMx system (Abbott Laboratories, Chicago, IL, USA), and folate concentrations were measured using a radioimmunoassay kit (ACS 180; Bayer, Tarrytown, NY, USA).

### Genotyping

DNA was extracted using the G-DEX blood extraction kit (iNtRON Biotechnology, Inc., Seongnam, Republic of Korea). The four best-studied SNPs in the *MTHFR* gene were determined by a documentary search, which included four 3′-UTR SNPs (2572 C > A, rs4846049; 4869 C > G, rs1537514; 5488 C > T, rs3737967; and 6685 T > C rs4846048). All SNP sequences were obtained from the HapMap database (http://www.hapmap.org). The *MTHFR* 2572 C > A and 4869 C > G polymorphisms were analyzed by the polymerase chain reaction-restriction fragment length polymorphism method. Real-time polymerase chain reaction (PCR) was used to analyze the *MTHFR* 5488 C > T and 6685 T > C polymorphisms. For each polymorphism, 30% of the PCR assay samples were randomly selected and repeated, and followed by DNA sequencing, to validate the RFLP findings. Sequencing was performed using an ABI 3730xl DNA Analyzer (Applied Biosystems, Foster City, CA, USA). The concordance of quality control samples was 100%.

### Statistical analyses

To analyze baseline characteristics, we used chi-square tests for categorical data and Student’s *t*-tests or analyses of variance were used for continuous data. We estimated associations of *MTHFR* gene polymorphisms with ischemic stroke incidence using adjusted ratios (AORs) and 95% confidence intervals (CIs) from multivariate logistic regression analyses. Adjustments were performed for sex, age, HTN, DM, hyperlipidemia, and smoking, because they are well-established risk factors for ischemic stroke. To evaluate the impact of *MTHFR* gene polymorphisms on all-cause mortality, we conducted hazard ratios (HRs), and 95% CIs from Kaplan-Meier survival analyses. Analyses were performed using GraphPad Prism 4.0 (GraphPad Software Inc., San Diego, CA, USA) and Medcalc version 12.7.1.0 (Medcalc Software, Mariakerke, Belgium). Haplotypes for multiple loci were estimated using the expectation-maximization algorithm with SNPAlyze (Version 5.1; DYNACOM Co. Ltd., Yokohama, Japan).

## Electronic supplementary material

Supplemental Dataset
