# Supplementary material for: Interplay between 3′-UTR polymorphisms in the methylenetetrahydrofolate reductase (MTHFR) gene and the risk of ischemic stroke
Source: Sci Rep. 2017 Sep 29;7:12464. doi: 10.1038/s41598-017-12668-x (PMC5622127; doi:10.1038/s41598-017-12668-x)
Supplement: Supplementary file 1 — Supplemental Dataset [file 41598_2017_12668_MOESM1_ESM.doc]

**Interplay between 3'-UTR polymorphisms in the methylenetetrahydrofolate reductase (MTHFR) gene and the risk of ischemic stroke**

Jung Oh Kim^1,2^, Hahn Sung Park^1,2^, Chang Soo Ryu^1,2^, Jung-Won Shin^3^, Jinkwon Kim^3^, Seung Hun Oh^3^, Ok Joon Kim,^3,†^ & Nam Keun Kim^1,2^,**^†^**

^1^Institute for Clinical Research, CHA Bundang Medical Center, School of Medicine, CHA University, Seongnam 463-712, South Korea; ^2^Department of Biomedical Science, College of Life Science, CHA University, Seongnam, South Korea; ^3^Department of Neurology, CHA Bundang Medical Center, School of Medicine, CHA University, Seongnam 463-712, South Korea

**Conflict of interest statement**

The authors have declared that no conflict of interest exists.

**^†^ Correspondence to**:

Nam Keun Kim, PhD

Institute for Clinical Research, School of Medicine, CHA University, 335, Pangyo-ro, Bundang-gu, Seongnam 463-400, South Korea.

Fax: +82-31-881-7249, TEL: +82-31-881-7137, E-mail: [nkkim@cha.ac.kr](mailto:nkkim@cha.ac.kr)

Ok Joon Kim, PhD

Department of Neurology, School of Medicine, CHA University,

351, Yatap-dong, Bundang-gu, Seongnam 463-712, South Korea.

Fax: +82-31-780-5766, TEL: +82-31-780-5762, E-mail: [nkkim@cha.ac.kr](mailto:nkkim@cha.ac.kr)

**Supplemental data**

| **Supplemental Table 1. Comparison of genotype frequencies and AOR of *MTHFR* gene polymorphisms between the ischemic stroke with/without undetermined subtypes, and control subjects** | | | | | | | | | |
| --- | --- | --- | --- | --- | --- | --- | --- | --- | --- |
| **Characteristics** | **Controls  (n=411)** | **Case without UD (n=409)** | **AOR(95% CI)*** | ***P^†^*** | ***P^‡^*** | **UD (n=102)** | **AOR(95% CI)*** | ***P^†^*** | ***P^‡^*** |
| ***MTHFR* 2572C>A** |  |  |  |  |  |  |  |  |  |
| CC | 280 (68.1) | 262 (64.1) | 1.000 (reference) |  |  | 71 (69.6) | 1.000 (reference) |  |  |
| CA | 122 (29.7) | 136 (33.3) | 1.297 (0.950-1.770) | 0.102 | 0.408 | 27 (26.5) | 0.962 (0.574-1.615) | 0.885 | 0.885 |
| AA | 9 (2.2) | 11 (2.7) | 1.493 (0.581-3.841) | 0.406 | 0.609 | 4 (3.9) | 1.975 (0.553-7.050) | 0.294 | 0.441 |
| Dominant ( CC vs. CA + AA ) |  |  | 1.314 (0.970-1.780) | 0.078 | 0.312 |  | 1.040 (0.633-1.710) | 0.876 | 0.876 |
| Recessive ( CC + CA vs. AA ) |  |  | 1.407 (0.552-3.582) | 0.474 | 0.686 |  | 2.139 (0.603-7.592) | 0.239 | 0.359 |
|  |  |  |  |  |  |  |  |  |  |
| ***MTHFR* 4869C>G** |  |  |  |  |  |  |  |  |  |
| CC | 368 (89.5) | 363 (88.8) | 1.000 (reference) |  |  | 94 (92.2) | 1.000 (reference) |  |  |
| CG | 43 (10.5) | 46 (11.2) | 1.145 (0.722-1.817) | 0.564 | 0.708 | 8 (7.8) | 0.819 (0.359-1.866) | 0.634 | 0.848 |
| GG | 0 (0.0) | 0 (0.0) |  |  |  | 0 (0.0) |  |  |  |
| Dominant ( CC vs. CG + GG ) |  |  | 1.145 (0.722-1.817) | 0.564 | 0.752 |  | 0.819 (0.359-1.866) | 0.634 | 0.876 |
| Recessive ( CC + CG vs. GG ) |  |  |  |  |  |  |  |  |  |
|  |  |  |  |  |  |  |  |  |  |
| ***MTHFR* 5488C>T** |  |  |  |  |  |  |  |  |  |
| CC | 351 (85.4) | 357 (87.3) | 1.000 (reference) |  |  | 93 (91.2) | 1.000 (reference) |  |  |
| CT | 59 (14.4) | 50 (12.2) | 0.922 (0.604-1.408) | 0.708 | 0.708 | 7 (6.9) | 0.525 (0.226-1.218) | 0.134 | 0.536 |
| TT | 1 (0.2) | 2 (0.5) | 1.632 (0.138-19.370) | 0.698 | 0.698 | 2 (2.0) | 7.644 (0.663-88.120) | 0.103 | 0.309 |
| Dominant ( CC vs. CT + TT ) |  |  | 0.939 (0.618-1.426) | 0.766 | 0.766 |  | 0.666 (0.310-1.430) | 0.297 | 0.876 |
| Recessive ( CC + CT vs. TT ) |  |  | 1.670 (0.139-20.026) | 0.686 | 0.686 |  | 9.071 (0.780-105.469) | 0.078 | 0.234 |
|  |  |  |  |  |  |  |  |  |  |
| ***MTHFR* 6685T>C** |  |  |  |  |  |  |  |  |  |
| TT | 326 (79.3) | 342 (83.6) | 1.000 (reference) |  |  | 84 (82.4) |  |  |  |
| TC | 83 (20.2) | 61 (14.9) | 0.783 (0.536-1.143) | 0.205 | 0.410 | 17 (16.7) | 0.864 (0.473-1.579) | 0.636 | 0.848 |
| CC | 2 (0.5) | 6 (1.5) | 3.367 (0.625-18.135) | 0.158 | 0.474 | 1 (1.0) | 1.679 (0.119-23.684) | 0.701 | 0.701 |
| Dominant ( TT vs. TC + CC ) |  |  | 0.841 (0.582-1.217) | 0.359 | 0.718 |  | 0.889 (0.492-1.608) | 0.698 | 0.876 |
| Recessive ( TT + TC vs. CC ) |  |  | 3.653 (0.686-19.449) | 0.129 | 0.387 |  | 1.890 (0.137-26.040) | 0.634 | 0.634 |
| Abbreviation; AOR, adjusted odd ratio; MTHFR, . *The adjusted odds ratio (AOR) on the basis of risk factors such as age, gender, hypertension, hyperlipidemia, diabetes mellitus, and smoking. ^†^The *P*-value calculated by multiple logistic regression on the basis of risk factors such as age, gender, hypertension, hyperlipidemia, and diabetes mellitus. ^‡^False discovery rate-adjusted P value for multiple hypothesis testing using the Benjamini-Hochberg method. | | | | | | | | | |

| **Supplemental Table 2. Stratified analyses of the *MTHFR* gene for sex, advanced age, hypertension, diabetes mellitus, hyperlipidemia, smoking, folate levels, and homocysteine levles.** | | | | | | | | |
| --- | --- | --- | --- | --- | --- | --- | --- | --- |
| Factor | *MTHFR* 2572CA+AA | | *MTHFR* 4869CG+GG | | *MTHFR* 5488CT | | *MTHFR* 6685TC | |
|  | AOR(95% CI)* | *P* | AOR(96% CI)* | *P* | AOR(97% CI)* | *P* | AOR(98% CI)* | *P* |
| Sex |  |  |  |  |  |  |  |  |
| male | 1.680 (1.073 - 2.632) | 0.023 | 2.537 (1.173 - 5.486) | 0.018 | 1.300 (0.680 - 2.487) | 0.427 | 0.607 (0.346 - 1.065) | 0.082 |
| female | 1.149 (0.801 - 1.648) | 0.451 | 0.725 (0.429 - 1.224) | 0.228 | 0.537 (0.323 - 0.894) | 0.017 | 0.930 (0.595 - 1.452) | 0.749 |
| Age (year) |  |  |  |  |  |  |  |  |
| < 63 | 1.333 (0.875 - 2.032) | 0.181 | 1.333 (0.700 - 2.539) | 0.382 | 0.837 (0.470 - 1.491) | 0.546 | 0.672 (0.388 - 1.165) | 0.157 |
| ≥63 | 1.452 (0.998 - 2.113) | 0.051 | 1.013 (0.574 - 1.788) | 0.964 | 0.762 (0.443 - 1.313) | 0.328 | 0.931 (0.593 - 1.461) | 0.754 |
| Hypertension |  |  |  |  |  |  |  |  |
| absent | 1.220 (0.822 - 1.813) | 0.324 | 1.035 (0.556 - 1.929) | 0.913 | 0.665 (0.378 - 1.171) | 0.157 | 0.767 (0.475 - 1.238) | 0.278 |
| present | 1.590 (1.068 - 2.368) | 0.022 | 1.288 (0.717 - 2.314) | 0.397 | 0.975 (0.551 - 1.723) | 0.930 | 0.875 (0.527 - 1.452) | 0.604 |
| Diabetes Mellitus |  |  |  |  |  |  |  |  |
| absent | 1.575 (1.160 - 2.138) | 0.004 | 1.168 (0.739 - 1.848) | 0.507 | 0.831 (0.542 - 1.274) | 0.395 | 0.931 (0.641 - 1.353) | 0.709 |
| present | 0.751 (0.385 - 1.464) | 0.400 | 1.015 (0.343 - 3.006) | 0.978 | 0.623 (0.229 - 1.691) | 0.353 | 0.383 (0.163 - 0.905) | 0.029 |
| Hyperlipidemia |  |  |  |  |  |  |  |  |
| absent | 1.432 (1.036 - 1.978) | 0.030 | 1.219 (0.753 - 1.971) | 0.421 | 0.872 (0.555 - 1.370) | 0.552 | 0.867 (0.579 - 1.299) | 0.489 |
| present | 1.370 (0.786 - 2.389) | 0.267 | 1.059 (0.439 - 2.555) | 0.898 | 0.590 (0.262 - 1.331) | 0.204 | 0.746 (0.386 - 1.445) | 0.385 |
| Smoking |  |  |  |  |  |  |  |  |
| no | 1.224 (0.872 - 1.717) | 0.243 | 0.790 (0.487 - 1.280) | 0.338 | 0.572 (0.360 - 0.911) | 0.019 | 0.845 (0.554 - 1.291) | 0.437 |
| yes | 1.820 (1.105 - 2.999) | 0.019 | 3.744 (1.348 - 10.403) | 0.011 | 1.884 (0.841 - 4.220) | 0.124 | 0.768 (0.418 - 1.411) | 0.396 |
| Folate (nmol/L)^a^ |  |  |  |  |  |  |  |  |
| > 3.16 | 1.494 (1.086 - 2.053) | 0.014 | 1.163 (0.729 - 1.856) | 0.526 | 0.818 (0.528 - 1.267) | 0.368 | 0.959 (0.650 - 1.414) | 0.832 |
| ≤3.16 | 1.574 (0.791 - 3.133) | 0.196 | 1.891 (0.519 - 6.888) | 0.334 | 1.317 (0.406 - 4.273) | 0.647 | 0.548 (0.238 - 1.260) | 0.157 |
| Hcy (μmol/L)^b^ |  |  |  |  |  |  |  |  |
| < 16.36 | 1.439 (1.037 - 1.997) | 0.030 | 1.011 (0.629 - 1.627) | 0.963 | 0.756 (0.485 - 1.180) | 0.219 | 1.046 (0.696 - 1.572) | 0.827 |
| ≥16.36 | 1.454 (0.839 - 2.520) | 0.182 | 3.053 (1.000 - 9.418) | 0.050 | 1.419 (0.552 - 3.650) | 0.468 | 0.476 (0.242 - 0.938) | 0.032 |
| Hcy, homocysteine. * CI indicates confidence interval. Adjusted by age, sex, hypertension, diabetes mellitus, hyperlipidemia, and smoking. ^a^ 3.16 nmol/L is based on the bottom 15% of folate levels in patients and controls. ^b^ 16.36 μmol/L is based on the top 15% of homocysteine levels in patients and controls. | | | | | | | | |

| **Supplemental Table 3. Comparison of genotype frequencies and AOR of *MTHFR* gene haplotype between the ischemic stroke subtypes by TOAST, and control subjects** | | | | | | | | | | |
| --- | --- | --- | --- | --- | --- | --- | --- | --- | --- | --- |
| Haplotypes | Controls (2n=822) | Stroke LAD (2n=402) | OR (95% CI) | *P* | Stroke SVD (2n=298) | OR (95% CI) | *P* | Stroke CE (2n=108) | OR (95% CI) | *P* |
| *MTHFR* 2572/4869/5488/6685 | | |  |  |  |  |  |  |  |  |
| C-C-C-T | 665 (81.0) | 319 (79.4) | 1.000 (reference) |  | 230 (77.2) | 1.000 (reference) |  | 76 (70.4) | 1.000 (reference) |  |
| A-C-C-T | 16 (1.9) | 27 (6.7) | 2.823 (1.559 - 5.110) | 0.001 | 18 (6.0) | 2.610 (1.357 - 5.021) | 0.004 | 9 (8.3) | 3.949 (1.736 - 8.983) | 0.001 |
| A-C-C-C | 67 (8.2) | 26 (6.5) | 0.877 (0.544 - 1.413) | 0.589 | 28 (9.4) | 1.310 (0.818 - 2.097) | 0.261 | 16 (14.8) | 2.265 (1.245 - 4.121) | 0.007 |
| A-G-T-T | 39 (4.7) | 23 (5.7) | 1.374 (0.799 - 2.364) | 0.251 | 19 (6.4) | 1.574 (0.883 - 2.807) | 0.124 | 5 (4.6) | 1.254 (0.477 - 3.296) | 0.647 |
| *MTHFR* 2572/4869/5488 | | | |  |  |  |  |  |  |  |
| C-C-C | 679 (82.6) | 319 (79.4) | 1.000 (reference) |  | 230 (77.2) | 1.000 (reference) |  | 77 (71.3) | 1.000 (reference) |  |
| A-C-C | 82 (10.0) | 53 (13.2) | 1.376 (0.950 - 1.992) | 0.091 | 46 (15.4) | 1.656 (1.120 - 2.448) | 0.011 | 25 (23.1) | 2.689 (1.621 - 4.459) | 0.0001 |
| A-G-T | 41 (5.0) | 23 (5.7) | 1.194 (0.705 - 2.024) | 0.510 | 20 (6.7) | 1.440 (0.827 - 2.509) | 0.198 | 5 (4.6) | 1.075 (0.413 - 2.803) | 0.882 |
| *MTHFR* 2572/4869/6685 | | | |  |  |  |  |  |  |  |
| C-C-T | 667 (81.1) | 321 (79.9) | 1.000 (reference) |  | 230 (77.2) | 1.000 (reference) |  | 76 (70.4) | 1.000 (reference) |  |
| A-C-T | 29 (3.5) | 29 (7.2) | 1.947 (1.153 - 3.286) | 0.013 | 19 (6.4) | 1.780 (0.986 - 3.213) | 0.056 | 10 (9.3) | 2.835 (1.338 - 6.010) | 0.007 |
| A-C-C | 71 (8.6) | 27 (6.7) | 0.826 (0.519 - 1.316) | 0.422 | 28 (9.4) | 1.196 (0.751 - 1.904) | 0.451 | 16 (14.8) | 2.068 (1.142 - 3.746) | 0.017 |
| A-G-T | 39 (4.7) | 24 (6.0) | 1.427 (0.835 - 2.440) | 0.194 | 20 (6.7) | 1.660 (0.939 - 2.933) | 0.081 | 5 (4.6) | 1.256 (0.478 - 3.301) | 0.644 |
| *MTHFR* 2572/5488/6685 | | | |  |  |  |  |  |  |  |
| C-C-T | 665 (81.0) | 320 (79.6) | 1.000 (reference) |  | 230 (77.2) | 1.000 (reference) |  | 76 (70.4) | 1.000 (reference) |  |
| A-C-T | 15 (1.9) | 28 (7.0) | 2.918 (1.619 - 5.260) | 0.0004 | 19 (6.4) | 2.755 (1.445 - 5.254) | 0.002 | 9 (8.3) | 3.949 (1.736 - 8.983) | 0.001 |
| A-C-C | 67 (8.2) | 26 (6.5) | 0.874 (0.543 - 1.408) | 0.580 | 28 (9.4) | 1.310 (0.818 - 2.097) | 0.261 | 16 (14.8) | 2.265 (1.245 - 4.121) | 0.007 |
| A-T-T | 52 (6.3) | 25 (6.2) | 1.133 (0.684 - 1.877) | 0.628 | 20 (6.7) | 1.261 (0.730 - 2.177) | 0.405 | 6 (5.6) | 1.145 (0.473 - 2.769) | 0.764 |
| *MTHFR* 4869/5488/6685 | | | |  |  |  |  |  |  |  |
| C-C-T | 681 (82.8) | 346 (86.1) | 1.000 (reference) |  |  | 1.000 (reference) |  |  | 1.000 (reference) |  |
| C-C-C | 80 (9.7) | 26 (6.5) | 0.698 (0.438 - 1.111) | 0.129 | 248 (83.2) | 1.048 (0.663 - 1.658) | 0.841 | 85 (78.7) | 1.857 (1.047 - 3.294) | 0.034 |
| C-T-T | 14 (1.7) | 4 (1.0) | 0.662 (0.212 - 2.067) | 0.478 | 28 (9.4) | 0.231 (0.030 - 1.785) | 0.160 | 17 (15.7) | 0.674 (0.087 - 5.245) | 0.706 |
| G-T-T | 41 (5.0) | 23 (5.7) | 1.269 (0.740 - 2.175) | 0.387 | 0 (0.0) | 1.462 (0.823 - 2.597) | 0.195 | 5 (4.6) | 1.123 (0.429 - 2.938) | 0.814 |
| Haplotypes of frequencies <5% and not significant were excluded. LAD, large artery disease; SVD, small vessel disease; CE, cardioembolism.  * *P*-values were calculated by Fisher's exact test. | | | | | | | | | | |

| **Supplemental Table 4.** **Analyses of variance for homocysteine levels according to combination model between *MTHFR* 677C>T and *MTHFR* 3'-UTR polymorphisms.** | | | | | | |
| --- | --- | --- | --- | --- | --- | --- |
|  | MTHFR 677CC | | MTHFR 677CT | | MTHFR 677TT | |
| Characteristics | Overall | | Overall | | Overall | |
|  | N | Mean±SD | N | Mean±SD | N | Mean±SD |
| *MTHFR* 2572CC | 143 | 9.57±4.07 | 330 | 10.04±4.40 | 166 | 13.28±7.19 |
| *MTHFR* 2572CA | 141 | 9.63±3.19 | 165 | 10.05±4.18 | 22 | 14.42±7.77 |
| *MTHFR* 2572AA | 23 | 12.14±5.56 | 2 | 8.27±0.81 | 2 | 23.37±19.30 |
| *P* |  | 0.010 |  | 0.846 |  | 0.134 |
| *MTHFR* 2572CA+AA | 164 | 9.98±3.7 | 167 | 10.03±4.16 | 24 | 15.16±8.81 |
| *P** |  | 0.356 |  | 0.981 |  | 0.246 |
| *MTHFR* 4869CC | 249 | 9.85±4.02 | 443 | 10.05±4.29 | 190 | 13.52±7.42 |
| *MTHFR* 4869CG | 54 | 9.28±3 | 53 | 9.97±4.65 | - | - |
| *MTHFR* 4869GG | 4 | 13.35±4.31 | 1 | 7.70 | - | - |
| *P* |  | 0.081 |  | 0.857 |  |  |
| *MTHFR* 4869CG+GG | 58 | 9.56±3.23 | 54 | 9.92±4.61 | - | - |
| *P** |  | 0.618 |  | 0.843 |  |  |
| *MTHFR* 5488CC | 239 | 9.94±4.1 | 434 | 10.07±4.30 | 189 | 13.54±7.43 |
| *MTHFR* 5488CT | 63 | 9.05±2.65 | 61 | 9.89±4.52 | 1 | 8.99 |
| *MTHFR* 5488TT | 5 | 12.04±4.74 | 2 | 7.63±0.10 | - | - |
| *P* |  | 0.114 |  | 0.701 |  | 0.542 |
| *MTHFR* 5488CT+TT | 68 | 9.27±2.9 | 63 | 9.82±4.46 | 1 | 8.99 |
| *P** |  | 0.209 |  | 0.668 |  | 0.542 |
| *MTHFR* 6685TT | 207 | 9.56±3.8 | 415 | 10.01±4.45 | 190 | 13.52±7.42 |
| *MTHFR* 6685TC | 90 | 9.78±3.34 | 82 | 10.15±3.63 | - | - |
| *MTHFR* 6685CC | 10 | 14.61±6.53 | 0 | - | - | - |
| *P* |  | < 0.001 |  | 0.797 |  |  |
| *MTHFR* 6685TC+CC | 100 | 10.27±4 | 82 | 10.15±3.63 | - | - |
| *P** |  | 0.136 |  | 0.797 |  |  |
| Data are presented as the mean ± standard deviation.  **P*-values were calculated using student t-tests. | | | | | | |


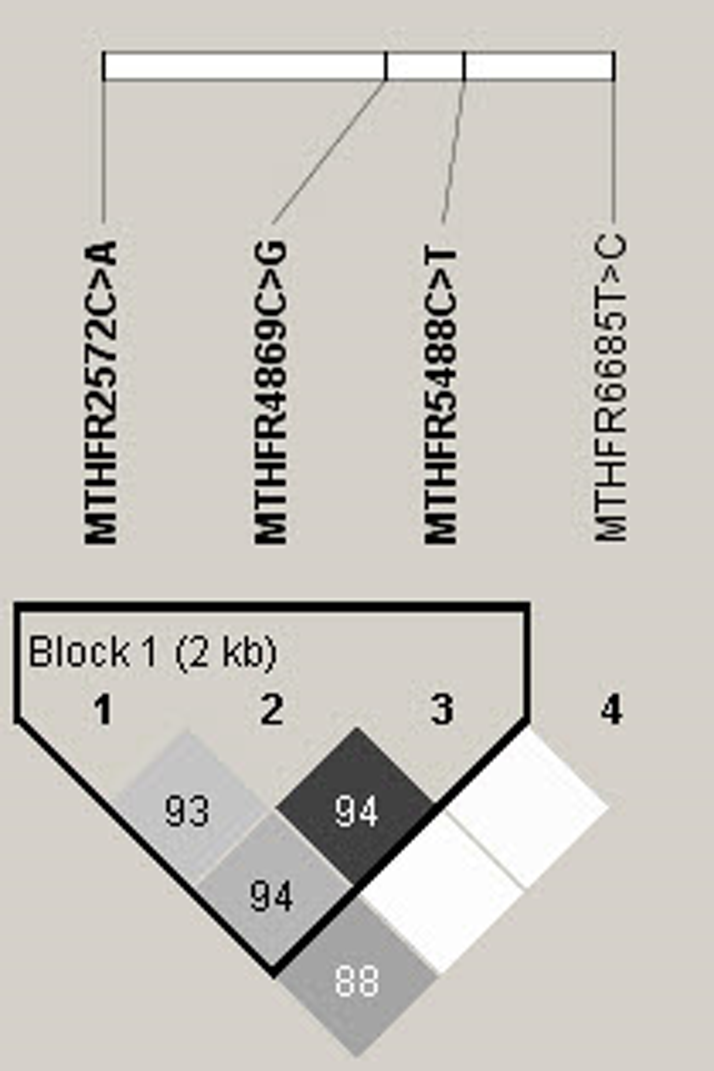


**Supplemental Figure 1. Linkage disequilibrium (LD) patterns of MTHFR SNPs.** Values in squares are LD between single markers. There were strong LDs between loci 2572C>A (rs4846049) and 4869C>G (rs1537514, D’=0.933), 4869C>G (rs1537514) and 5488C>T (rs3737967, D’=0.944), 2572C>A (rs4846049) and 5488C>T (rs3737967, D’=0.940) in ischemic stroke subjects. Dark squares indicate high r2 and bright squares indicate low r^2^ values.


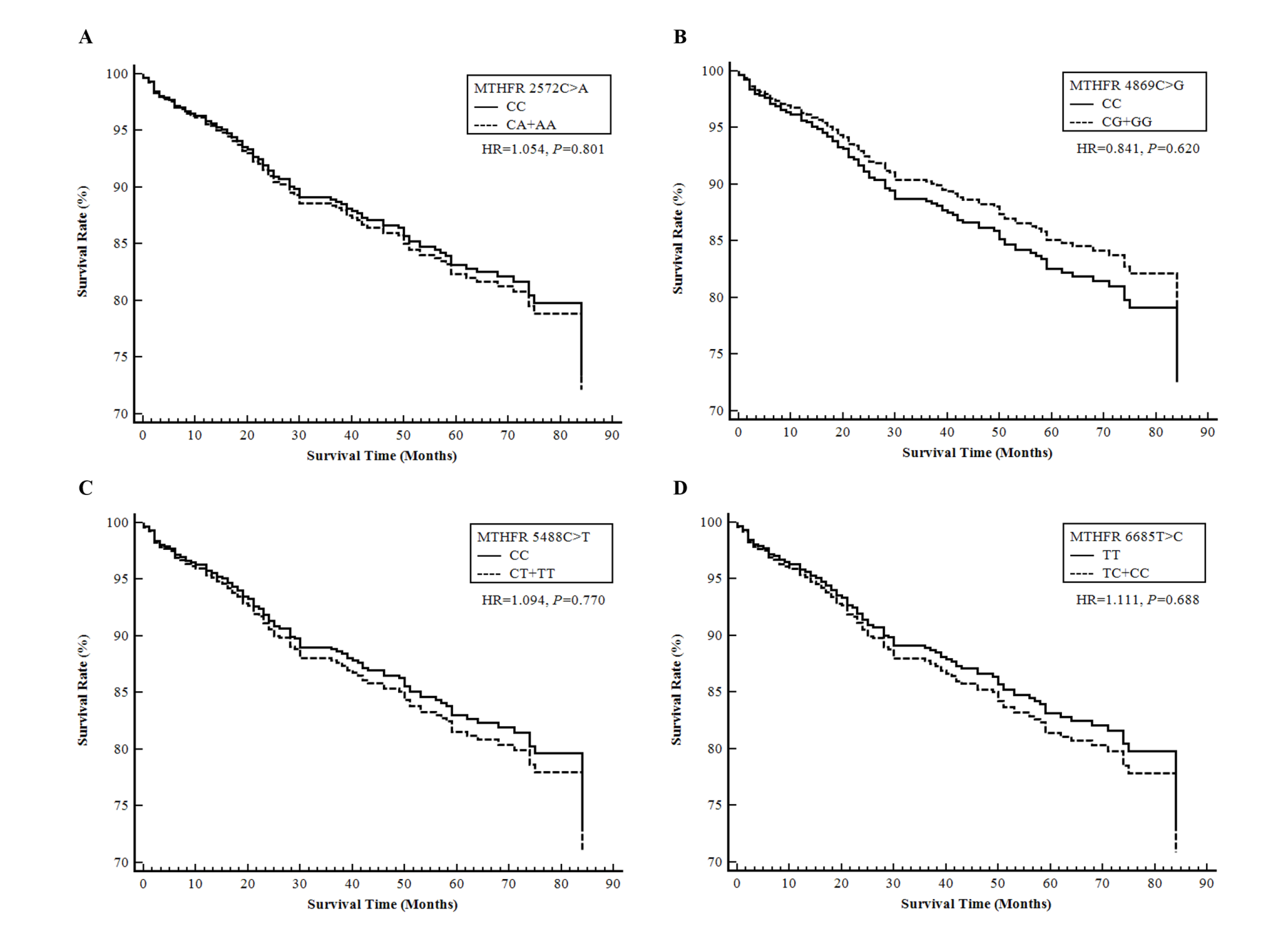


**Supplemental Figure 2. Overall survival (OS) rates of MTHFR 3'-UTR polymorphisms.** (A) OS curve of patients with MTHFR 2572CA+AA compared to patients with MTHFR 2572CC. (B) OS curve of patients with MTHFR 4869CG+GG compared to patients with MTHFR 4869CC. (C) OS curve of patients with MTHFR 5488CT+TT compared to patients with MTHFR 5488CC. (D) OS curve of patients with MTHFR 6685TC+CC compared to patients with MTHFR 6685TT.
